# The association of cardiorespiratory fitness with spirometric small airway obstruction

**DOI:** 10.1183/23120541.00275-2023

**Published:** 2023-08-29

**Authors:** Ben Knox-Brown, Karl Sylvester, Andre F.S. Amaral

**Affiliations:** 1 National Heart and Lung Institute, Imperial College London, London, UK; 2 Respiratory Physiology, Papworth Hospital NHS Foundation Trust, Cambridge, UK; 3 Respiratory Physiology, Cambridge University Hospitals NHS Foundation Trust, Cambridge, UK; 4 NIHR Imperial Biomedical Research Centre, London, UK

## Abstract

Spirometric small airway obstruction is commonly assessed using the mean forced expiratory flow rate between 25% and 75% of the forced vital capacity (FEF_25–75%_) [1]. It has a global prevalence ranging from 5% to 34%, is associated with risk factors like those of COPD [2], and is prognostic of future chronic airflow obstruction [3]. Despite this, its clinical application has been limited. Recently, McNeill *et al.* [4] showed that the forced vital capacity (FVC) is associated with exercise capacity. Little is known regarding the impact of spirometric small airway obstruction on markers of cardiorespiratory fitness. We aimed to investigate this association using data from cardiopulmonary exercise tests (CPETs).

## Tweetable abstract

**Spirometric small airway obstruction is associated with impaired ventilatory response to exercise independently of FEV_1_/FVC ratio** https://bit.ly/3pre4sk

*To the Editor:*

Spirometric small airway obstruction is commonly assessed using the mean forced expiratory flow rate between 25% and 75% of the forced vital capacity (FEF_25–75%_) [1]. It has a global prevalence ranging from 5% to 34%, is associated with risk factors like those of COPD [2], and is prognostic of future chronic airflow obstruction [3]. Despite this, its clinical application has been limited. Recently, McNeill *et al.* [4] showed that the forced vital capacity (FVC) is associated with exercise capacity. Little is known regarding the impact of spirometric small airway obstruction on markers of cardiorespiratory fitness. We aimed to investigate this association using data from cardiopulmonary exercise tests (CPETs).

We collected data from patients referred to Cambridge University Hospitals NHS Foundation Trust, Cambridge, UK, for spirometry and CPET between January 2016 and May 2021. Data were included if both measurements were taken on the same day, spirometry was acceptable according to American Thoracic Society/European Respiratory Society standards [5] and maximal effort was given during the CPET according to predefined criteria [6]. CPETs were performed on a ramp-incremental cycle ergometer using the Vyntus CPX Metabolic Cart. Patients performed baseline spirometry, followed by maximal-effort incremental cycling at 60–63 rpm. The work rate protocol (in Watts per minute) was calculated according to height, weight, age and sex. We used multivariable linear regression to assess the association of continuous CPET measurements, with spirometric small airway obstruction, defined as FEF_25–75%_ (in litres per second) less than the lower limit of normal (LLN). We then used multivariable logistic regression to investigate the association of binary CPET measurements (peak oxygen uptake (*V*′_O_2__) <80% predicted, peak oxygen pulse <80% predicted, peak breathing reserve <15 L·min^−1^ or 15% of maximum predicted ventilation (calculated as 35×forced expiratory volume in 1 s (FEV_1_)) without cardiovascular limitation, which we defined as having a heart rate reserve ≤15 beats per min) with spirometric small airway obstruction [7]. Regression analyses were adjusted for age, sex, body mass index, smoking status, referral reason and FVC. p-values were adjusted for multiple testing using the Bonferroni correction, with significance set at p<0.05. Data were analysed using Stata 17 (Stata Corp., College Station, TX, USA). We performed secondary analyses for isolated spirometric small airway obstruction defined as FEF_25–75%_<LLN with a normal FEV_1_/FVC ratio (≥LLN), and sensitivity analysis on those referred for unexplained breathlessness. Due to the wide limits of normal for FEF_25–75%_ and its close association with the FVC [8], we re-ran the analyses defining spirometric small airway obstruction using the forced expiratory volume in 3 s (FEV_3_)/FVC ratio, which has been independently associated with clinical markers of small airway disease [9]. We used established reference equations for spirometry [10, 11] and CPET [12]. This study was approved by the Health Research Authority (IRAS project ID 301467).

We collected data from 419 patients. After excluding those with poor quality spirometry (n=11) and with physiologically submaximal CPET (no plateau in *V*′_O_2__, and no evidence of cardiovascular or ventilation limitation at peak exercise; n=186), our dataset included 222 patients. The mean±sd age was 51.8±15.6 years with 115 (52%) being female. Most patients were never- (54%) or former smokers (44%). Reasons for referral included 133 (62%) patients with unexplained breathlessness, who had normal spirometry at time of referral; 36 (17%) with asthma; seven (3%) with COPD; four (2%) with bronchiectasis; four (2%) with sarcoidosis; five (2%) with query cardiac abnormality; five (2%) with chronic cough of unknown cause; 13 (6%) for pre-operative assessment; five (2%) with post COVID-19 shortness of breath; and one (0.5%) with pulmonary vascular disease. For FEF_25–75%_, spirometric small airway obstruction was present in 38 (17%) patients, with isolated spirometric small airways obstruction present in 17 (9%) patients; while for FEV_3_/FVC, 30 (14%) patients had spirometric small airway obstruction and 13 (7%) had isolated spirometric small airway obstruction. For those referred for unexplained breathlessness, isolated spirometric small airway obstruction was present in 12 (11%) and eight (7%) patients for FEF_25–75%_ and FEV_3_/FVC respectively. Mean peak *V*′_O_2__ was 98.95±26.48% predicted, with 59 (27%) patients having a low peak *V*′_O_2__. Mean peak oxygen pulse was 98.35±27.00% predicted, with 56 (25%) patients reporting a low end-exercise oxygen pulse. Mean peak breathing reserve was 19.42±22.98 L·min^−1^, with ventilatory limitation seen in 41 (18%) patients. Spirometric small airway obstruction for FEF_25–75%_<LLN was associated with a lower end-exercise breathing reserve (β= −22.10 L·min^−1^, 95% CI −29.81– −14.37 L·min^−1^; p<0.001), greater odds of ventilation limitation at peak exercise (OR 17.5, 95% CI 5.71–53.5; p=0.001) but not low peak *V*′_O_2__ or end-exercise oxygen pulse. Having isolated spirometric small airway obstruction was also associated with lower breathing reserve (β= −18.14 L·min^−1^, 95%CI −28.65– −7.64 L·min^−1^; p=0.001) and ventilatory limitation (OR 32.7, 95% CI 5.82–183; p<0.001). Associations were the same when spirometric small airways obstruction was defined using the FEV_3_/FVC ratio (table 1), with greater odds of ventilatory limitation for spirometric small airway obstruction (OR 3.39, 95% CI 1.28–8.94; p=0.014) and isolated spirometric small airway obstruction (OR 6.81, 95% CI 1.60–28.9; p=0.009). Spirometric restriction was associated with greater odds of low peak *V*′_O_2__ (OR 2.52, 95% CI 1.05–6.05; p=0.039), end-exercise oxygen pulse (OR 2.57, 95% CI 1.05–6.28; p=0.038) and ventilatory limitation (OR 3.02, 95% CI 1.18–7.71; p=0.021). Airflow obstruction was associated with ventilatory limitation only (OR 4.39, 95% CI 1.74–11.1; p=0.002). Among those referred for unexplained breathlessness, spirometric small airway obstruction using FEF_25–75%_ was the only spirometry parameter associated with a reduced breathing reserve (β= −19.94 L·min^−1^, 95% CI −29.84– −10.05 L·min^−1^, p<0.001).

**TABLE 1:** Linear association of cardiopulmonary exercise test (CPET) variables with spirometric small airway obstruction, airflow obstruction and spirometric restriction

	**Spirometric** **small airway obstruction,** **FEF_25–75%_<LLN^#^**		**Spirometric** **small airway obstruction,** **FEV_3_/FVC<LLN^¶^**		**Spirometric restriction,** **FVC<LLN^+^**		**Airflow obstruction, FEV_1_/FVC<LLN^§^**	
	**β (95%CI)**	**p-value**	**β (95%CI)**	**p-value**	**β (95%CI)**	**p-value**	**β (95%CI)**	**p-value**
**Peak *V*′_O_2__**, **mL·kg^−1^ ·min^−1^**	−1.84 (−4.05–0.37)	0.102	−0.44 (−2.91–2.03)	0.727	−5.36 (−7.60– −3.12)	<0.001*	−0.22 (−2.39–1.96)	0.844
**Peak *V*′_O_2__, % pred**	−7.12 (−16.46–2.21)	0.134	5.37 (−5.01–15.75)	0.309	−17.96 (−27.59– −8.32)	<0.001*	0.41 (−8.76–9.59)	0.930
***V*′_O_2__ at AT, mL·kg^−1^ ·min^−1^**	0.34 (−1.14–1.84)	0.649	0.13 (−1.53–1.79)	0.878	−2.33 (−3.88– −0.79)	0.003*	0.12 (−1.34–1.59)	0.868
**End-exercise oxygen pulse, *V*′_O_2__/HR**	−0.20 (−1.30–0.90)	0.716	0.16 (−1.05–1.38)	0.793	−1.15 (−2.31–0.00)	0.050	0.45 (−0.62–1.52)	0.412
**Cardiovascular slope, ΔHR/Δ*V*′_O_2__**	1.71 (−3.95–7.37)	0.552	−1.15 (−7.40–5.11)	0.717	2.06 (−3.82–7.94)	0.491	−5.90 (−11.26– −0.54)	0.031
**End-exercise *V*′_E_, L·min^−1^**	−7.37 (−14.73– −0.01)	0.050	0.67 (−7.56–8.91)	0.872	−12.74 (−20.43– −5.06)	0.001*	0.39 (−6.87–7.65)	0.916
**Peak *V*′_E_, % pred**	17.96 (11.12–24.80)	<0.001*	13.52 (5.67–21.39)	0.001*	5.75 (−1.95–13.46)	0.143	12.44 (5.53–19.36)	<0.001*
**Breathing reserve at peak exercise, L·min^−1^**	−22.10 (−29.81– −14.37)	<0.001*	−13.3 (−22.34– −4.24)	0.004*	−9.54 (−18.29– −0.78)	0.033	−13.40 (−21.32– −5.47)	0.001*
**Breathing reserve, %**	−18.10 (−24.95– −11.23)	<0.001*	−13.46 (−20.35– −5.56)	0.001*	−7.72 (−14.34– –1.10)	0.022	−12.44 (−19.38– −5.51)	0.001*
**ΔIC, mL**	−383.17 (−559.94– −206.42)	<0.001*	−348.99 (−548.02– −149.96)	0.001*	−116.78 (−285.56–51.99)	0.174	−197.67 (−379.13– −16.20)	0.033
***V*′_E_/*V*′_CO_2__ slope**	−0.86 (−7.60–5.87)	0.801	0.80 (−6.67–8.26)	0.833	−0.77 (−7.92–6.37)	0.832	−0.08 (−6.67–6.50)	0.980
**Δ*S*_pO_2__, %**	0.53 (−0.30–1.38)	0.211	0.57 (−0.37–1.51)	0.231	0.64 (−0.26–1.53)	0.160	0.76 (−0.06–1.58)	0.069
**Eq*V*** **′_O_2__ at AT**	1.87 (0.06–3.68)	0.042	1.49 (−0.52–3.51)	0.144	0.82 (−1.12–2.76)	0.404	1.13 (−0.64–2.91)	0.212
**Eq*V*′_CO_2__ at AT**	1.33 (−0.27–2.93)	0.103	1.39 (−0.39–3.17)	0.124	1.51 (−0.19–3.22)	0.080	1.09 (−0.49–2.66)	0.174
**Borg score at rest**	0.26 (−0.10–0.61)	0.146	0.48 (0.08–0.87)	0.018	0.10 (−0.26–0.45)	0.593	0.38 (0.04–0.71)	0.028
**Borg score at peak**	0.60 (−0.21–1.41)	0.143	0.47 (−0.43–1.37)	0.303	−0.33 (−1.17–0.50)	0.431	0.44 (−0.34–1.22)	0.270

Results interpreted as the change in the CPET parameter for each spirometric condition. The spirometry lower limit of normal (LLN) was derived from the National Health and Nutrition Examination Survey reference equations. CPET reference equations were taken from the SHIP study. Analyses were adjusted for sex, age, body mass index, smoking status (never- (ref.)/former/current) and referral reason (unexplained breathlessness (ref.)/COPD/asthma/bronchiectasis/sarcoidosis/query cardiac abnormality/unexplained chronic cough/pre-operative/post-COVID-19). Data were considered significant if p≤0.05. FEF_25–75%_: mean forced expiratory flow rate between 25% and 75% of the forced vital capacity; FEV*_x_*: forced expiratory volume in *x* s; FVC: forced vital capacity; *V*′_O_2__: oxygen uptake; AT: anaerobic threshold; HR: heart rate; Δ: change from pre- to peak exercise; *V*′_E_: minute ventilation; IC: inspiratory capacity; *V*′_CO_2__: carbon dioxide output; *S*_pO_2__: oxygen saturation measured by pulse oximetry; Eq: ventilatory equivalent for. ^#^: n=38; ^¶^: n=30; ^+^: n=33; ^§^: n=40. *: result significant after Bonferroni correction for multiple testing.

Our study shows that impaired ventilatory response to exercise is associated with spirometric small airway obstruction measured using either FEF_25–75%_ or FEV_3_/FVC. Furthermore, we have shown that patients with isolated spirometric small airway obstruction are more likely to experience ventilation limitation at peak exercise. A potential explanation is that spirometric small airway obstruction identifies individuals with mild airway disease not detected by traditional measurement indices, which impairs ventilatory response to maximal exercise. Our results contrast with those of Holley *et al.* [13], who found no association between isolated spirometric small airway obstruction and any ventilatory parameters, which could be explained by their smaller sample size and low number of cases. We found no association between spirometric small airway obstruction and *V*′_O_2__ at the anaerobic threshold, peak *V*′_O_2__ or end-exercise oxygen pulse, suggesting, particularly in the case of an isolated abnormality, that obstruction may be too mild to impact submaximal and maximal markers of cardiorespiratory fitness. In addition, like McNeill *et al.* [4], we found that the FVC was the only spirometry parameter associated with these parameters. End-exercise oxygen pulse has been suggested as a noninvasive marker of stroke volume [14], which supports previous research showing that spirometric restriction may increase the risk of cardiovascular disease [15]. Of particular interest was our finding that only FEF_25–75%_<LLN was associated with a lower breathing reserve at peak exercise in those with unexplained breathlessness, suggesting that FEF_25–75%_ may be important in this population.

Our study has limitations. First, our sample size was affected by the exclusion of a high number of physiologically submaximal exercise tests. A further limitation is that our findings cannot be easily applied to the general population and further studies in general populations are needed to confirm our observations.

In conclusion, spirometric small airway obstruction was not associated with overall cardiorespiratory fitness. However, it was associated with impaired ventilatory response to exercise. This provides further evidence in support of the recognition of spirometric small airways obstruction as a genuine physiological phenomenon.
